# SSVEP BCI and Eye Tracking Use by Individuals With Late-Stage ALS and Visual Impairments

**DOI:** 10.3389/fnhum.2020.595890

**Published:** 2020-11-20

**Authors:** Betts Peters, Steven Bedrick, Shiran Dudy, Brandon Eddy, Matt Higger, Michelle Kinsella, Deirdre McLaughlin, Tab Memmott, Barry Oken, Fernando Quivira, Scott Spaulding, Deniz Erdogmus, Melanie Fried-Oken

**Affiliations:** ^1^Consortium for Accessible Multimodal Brain-Body Interfaces (CAMBI), Portland, OR, United States; ^2^REKNEW Projects, Institute on Development & Disability, Department of Pediatrics, Oregon Health & Science University, Portland, OR, United States; ^3^Department of Medical Informatics and Clinical Epidemiology, Oregon Health & Science University, Portland, OR, United States; ^4^Center for Spoken Language Understanding, Institute on Development and Disability, Department of Pediatrics, Oregon Health & Science University, Portland, OR, United States; ^5^Khoury College of Computer Science, Northeastern University, Boston, MA, United States; ^6^Department of Neurology, Oregon Health & Science University, Portland, OR, United States; ^7^Independent researcher, Boston, MA, United States; ^8^College of Education, University of Washington, Seattle, WA, United States; ^9^Cognitive Systems Laboratory, Center for Signal Processing, Imaging, Reasoning, and Learning (SPIRAL), Department of Electrical and Computer Engineering, Northeastern University, Boston, MA, United States

**Keywords:** augmentative and alternative communication, vision disorders, brain-computer interface, steady state visual evoked potential, eye tracking, amyotrophic lateral sclerosis

## Abstract

Access to communication is critical for individuals with late-stage amyotrophic lateral sclerosis (ALS) and minimal volitional movement, but they sometimes present with concomitant visual or ocular motility impairments that affect their performance with eye tracking or visual brain-computer interface (BCI) systems. In this study, we explored the use of modified eye tracking and steady state visual evoked potential (SSVEP) BCI, in combination with the Shuffle Speller typing interface, for this population. Two participants with late-stage ALS, visual impairments, and minimal volitional movement completed a single-case experimental research design comparing copy-spelling performance with three different typing systems: (1) commercially available eye tracking communication software, (2) Shuffle Speller with modified eye tracking, and (3) Shuffle Speller with SSVEP BCI. Participant 1 was unable to type any correct characters with the commercial system, but achieved accuracies of up to 50% with Shuffle Speller eye tracking and 89% with Shuffle Speller BCI. Participant 2 also had higher maximum accuracies with Shuffle Speller, typing with up to 63% accuracy with eye tracking and 100% accuracy with BCI. However, participants’ typing accuracy for both Shuffle Speller conditions was highly variable, particularly in the BCI condition. Both the Shuffle Speller interface and SSVEP BCI input show promise for improving typing performance for people with late-stage ALS. Further development of innovative BCI systems for this population is needed.

## Introduction

Amyotrophic lateral sclerosis (ALS) is a progressive neurodegenerative disease that affects voluntary motor function. The course of disease progression and life expectancy vary considerably from person to person, but a majority of people with ALS (PALS) lose the ability to speak, and thus may benefit from augmentative and alternative communication (AAC) approaches (Beukelman et al., 2011; Fried-Oken et al., 2015a). Loss of function in the upper extremities often necessitates the use of alternative access methods (Gibbons and Beneteau, 2010). Since most PALS maintain adequate ocular motility even as other voluntary motor function deteriorates, they are often prescribed eye tracking speech-generating devices (SGDs), which allow them to type messages using only eye movements. However, in the later stages of ALS, ocular motility may be impaired, the eyes may become dry due to poor eyelid function, or ptosis (drooping of the eyelids) may partially obscure the pupil (Hayashi and Oppenheimer, 2003; Ball et al., 2010; Moss et al., 2012; Spataro et al., 2014; Nakayama et al., 2016). Any of these factors may reduce eye tracking accuracy or effectiveness and therefore make typing with AAC software difficult or impossible (Ball et al., 2010; Spataro et al., 2014; Chen and O’Leary, 2018). Other visual conditions, such as reduced visual acuity, diplopia, or light sensitivity, may not affect eye tracking but may impair the ability to see or interact with a visual interface (Fried-Oken et al., 2020). Furthermore, PALS may avoid or limit use of eye tracking SGDs due to fatigue associated with eye movement (Spataro et al., 2014). Some PALS, particularly those who elect to undergo tracheostomy and receive invasive mechanical ventilation, may eventually progress to a completely locked-in state, in which all voluntary motor function, including eye movement, is lost (Hayashi and Oppenheimer, 2003; Murguialday et al., 2011; Nakayama et al., 2016).

Brain-computer interface (BCI) technology, which allows computer control without voluntary motor function, offers a potential communication access method for individuals who are locked in due to late-stage ALS or other conditions (Akcakaya et al., 2014). Although researchers have introduced BCI systems using auditory and tactile stimulation, the majority of BCIs rely on a visual user interface. As with eye tracking SGDs, visual and oculomotor impairments may limit PALS’s performance with visual BCI systems (Fried-Oken et al., 2020). Although this potential association has not been empirically explored, one study of P300 BCI use by PALS classified participants into successful and unsuccessful users based on their performance results, and found that all members of the unsuccessful group presented with visual impairment (including diplopia, nystagmus, or ptosis) (McCane et al., 2014). Other research has explored the impact of simulated ocular motility impairments on BCI performance for healthy control participants. In such studies, the popular P300 matrix speller has been found to rely heavily on overt visual attention and eye movement (Brunner et al., 2010; Treder and Blankertz, 2010; Riccio et al., 2012), though alternative visual interfaces have led to improvements in performance with covert attention (Treder and Blankertz, 2010; Treder et al., 2011).

Previous work by the authors introduced the Shuffle Speller typing interface, designed to adapt to user input characteristics by adjusting stimulus presentation based on each individual’s unique pattern of responses and errors (Higger et al., 2016). In an earlier study, Shuffle Speller was reconfigured to accommodate acuity and ocular motility impairments, with large font sizes, high contrast, and color and contextual cues. Healthy control participants used this version of Shuffle Speller with a steady state visual evoked potential (SSVEP) BCI under conditions simulating reduced visual acuity and impaired ocular motility (Peters et al., 2018a). All participants were able to use the system with a simulated visual acuity impairment of 20/200 (the level associated with legal blindness in the United States), with no significant effect on typing accuracy or speed compared to an unimpaired control condition. Performance under a simulated ocular motility impairment condition was more variable, with only a small number of participants (6/37) able to type successfully. For participants who were successful under this condition, typing accuracy was comparable to performance under the unimpaired condition, but speed was greatly reduced, reflecting Shuffle Speller’s adaptations (increased trial length and more trials per decision) to more ambiguous user input signals.

In this work, we describe a new experiment to explore the use of Shuffle Speller, with eye tracking and SSVEP BCI, by individuals with late-stage ALS who have minimal volitional movement, impaired ocular motility, and other concomitant visual challenges. Individuals with this level of impairment would be classified as stage 4 according to both the King’s clinical staging scale (nutritional or respiratory failure) and the MiToS ALS functional staging scale (loss of independence in the four domains of the ALSFRS-R) (Roche et al., 2012; Chiò et al., 2015), and may present with incomplete, classic, or total locked-in syndrome. This experiment serves as a feasibility study demonstrating the potential of the Shuffle Speller interface, as well as SSVEP BCI, to support communication by individuals with ALS presenting with locked-in syndrome.

Initially, a pilot study was conducted to investigate the feasibility of Shuffle Speller as an AAC interface in this population. Three PALS with poor eye tracking SGD performance were included as participants. Two of the three showed marked improvement in typing accuracy under the Shuffle Speller conditions, with both eye tracking and SSVEP BCI, compared to baseline performance with a commercial eye tracking SGD. The third showed reduced variability in performance and a higher overall mean accuracy for Shuffle Speller eye tracking compared to baseline (Peters, 2019).

For the current study, an alternating-treatments single-case experimental research design was used to assess whether Shuffle Speller, accessed with either SSVEP BCI or modified eye tracking, allowed more accurate typing than a similar typing interface accessed with traditional eye tracking for individuals with ALS and visual impairments.

## Materials and Methods

### Participants

Participants included two individuals with late-stage ALS. Both had participated in the pilot study described above, and were originally recruited through prior contact with the research team or through their speech-language pathologist (SLP). They met the following inclusion criteria: (1) diagnosed with ALS by a neurologist, (2) able to respond reliably to yes/no questions, and (3) unable to use commercially available eye tracking hardware and AAC software for spelling, by self-report or the report of an SLP. Participant 1 had successfully controlled an eye tracking SGD for many years, using it for several hours each day to type messages on a QWERTY onscreen keyboard as well as to access a web browser, email client, and other applications. However, eye tracking became increasingly unreliable as his ALS progressed, and he stopped using the SGD altogether approximately 4 years before the study began. Participant 2 had trialed eye tracking with an experienced SLP, but the trials were unsuccessful, and he instead accessed his SGD using a proximity switch activated with small thumb movements. Both participants demonstrated a reliable yes/no response at the beginning of the study, though Participant 1’s signals became less consistent and more difficult to observe over the course of the experiment. Participant 1 participated in a prior BCI study involving the RSVP Keyboard, a P300-based, rapid serial visual presentation spelling interface (Oken et al., 2014), and demonstrated poor calibration and spelling performance. Participant 2 had used the RSVP Keyboard in a different earlier study with high accuracy (Fried-Oken et al., 2014).

Both participants presented with an ALSFRS-R score of 0. Each demonstrated minimal volitional movements, such as slight jaw extension and retraction, minute thumb extensions, or lateral eye movements with significantly reduced range of motion. As such, they would be classified as having incomplete locked-in syndrome (Bauer et al., 1979). However, the strength and consistency of these remaining motor functions for both participants were observed to deteriorate over the course of the experiment, indicating that both were progressing toward total locked-in syndrome.

Each participant completed the Revised BCI Sensory/Cognitive/Communication Screen (Fried-Oken et al., 2015b; Peters et al., 2018b) which includes questions and tasks designed to screen vision, hearing, communication, cognitive, and literacy skills relevant to BCI use, as well as medications and motor function. The screen is intended for use with people with severe speech and physical impairment (SSPI), with responses given via yes/no signals or eye movements. Both participants gave consent for the review of results from their most recent eye examination, which were considered along with results from the vision section of the screen. Information on participant demographics, ALS Functional Rating Scale-Revised (ALSFRS-R) scores, physical function, visual skills and impairments, and communication methods is presented in Table 1.

**TABLE 1:** Participant demographics, visual skills, and communication methods.

	**Participant 1**	**Participant 2**
**Age (years)**	45	67
**Gender**	Male	Male
**Education (years)**	23	18
**Time since diagnosis (years)**	12	7
**ALSFRS-R score**	0	0
**Physical function**	Eye and facial movements only	Eye, facial, and left thumb movements only
**Glasses type**	Prescription lenses for distance viewing; prismatic lenses for near task viewing	Inverted bifocals (near viewing at top of lenses, distance viewing at bottom) with tinting to reduce light sensitivity
**Distance visual acuity (both eyes, corrected)**	20/30	20/60
**Near visual acuity (both eyes, corrected)**	20/60	20/90
**Visual impairments**	Endorsed “trouble seeing up close” Ptosis Reduced ability and accuracy for pursuit and saccade movements in both eyes Reduced visual figure-ground perceptual skills Alternating exotropia for near vision Convergence insufficiency History of corneal ulcer	Light sensitivity Reduced clarity at near viewing distance Nuclear sclerotic cataracts both eyes Intermittent double vision Ptosis
**Communication method(s)**	Yes/no responses (facial movements; increasingly unreliable as the study progressed) Partner-assisted scanning	Yes/no responses (eye movements) Single-switch scanning on SGD (thumb movements with proximity switch)

This study was approved by the Oregon Health & Science University (OHSU) Institutional Review Board (approval #15331), and participants gave informed consent. Following procedures outlined by Vansteensel et al. (2016) and incorporated into the Revised BCI Sensory/Cognitive/Communication Screen (Peters et al., 2018b) participants reviewed informed consent documents and answered yes/no questions to confirm their understanding. They then instructed an authorized research representative (a relative or paid caregiver) to sign the consent form on their behalf.

### Study Design

The experiment used an alternating treatments single-case experimental research design, including a baseline phase, replicated across participants. These designs allow relatively rapid comparison of two or more interventions (Barlow and Hayes, 1979) with repeated measurement of reversible target behaviors to evaluate differences in performance between conditions. They also allow exploration of individual variations in performance (Krasny-Pacini and Evans, 2018). The relatively short duration of alternating treatments designs can control for threats to internal validity such as maturation or instrumentation effects (Wolery and Gast, 2018). Inclusion of a baseline phase describes pre-intervention performance. In this case, the baseline phase featured a copy-spelling task designed to represent performance with existing eye tracking SGD technology, using a page set designed in Communicator 5 software (Tobii Dynavox, Pittsburgh, PA, United States) and a 1.2 s dwell time for selection (C1.2). (This is the longest of the three default dwell time options offered in Communicator 5 for typical eye tracking setup). Because of the slow and restricted eye movements observed in our target population, we wished to explore whether a longer dwell time of 2.5 s would improve performance by allowing participants more time to move their gaze between targets prior to selection, so this was included as a condition in the comparison phase. The comparison phase alternated copy-spelling tasks using three different typing systems: (1) a version of the Communicator 5 system, modified with an extended dwell time of 2.5 s (C2.5), (2) Shuffle Speller accessed with eye tracking (SSET), and (3) Shuffle Speller accessed with SSVEP BCI (SSBCI). Conditions are summarized in Table 2 and described below. Condition order in the comparison phase was randomly assigned, with no more than two consecutive sessions of the same condition as required by SCRD standards (What Works Clearinghouse, 2014; Wolery and Gast, 2018). Conditions were easily discriminable due to obvious differences among the user interfaces (e.g., different calibration procedures and the presence or absence of animated letters or flashing lights), and participants were specifically alerted to the current condition by an instructional video shown before each task (see Supplementary Materials).

**TABLE 2:** Experimental conditions, variables, and tasks.

**Phase**	**Baseline**	**Comparison**	**Comparison**	**Comparison**

**Condition**	Communicator 1.2 (C1.2)	Communicator 2.5 (C2.5)	Shuffle Speller Eye Tracking (SSET)	Shuffle Speller SSVEP BCI (SSBCI)

**Independent variable state (typing system)**	3-step speller in Communicator 5 with standard Tobii eye tracking and 1.2-second dwell time	3-step speller in Communicator 5 with standard Tobii eye tracking and 2.5-second dwell time	Shuffle Speller with modified eye tracking	Shuffle Speller with SSVEP BCI

**Primary dependent variable**	Typing accuracy (% correct character selections/total selections)	Typing accuracy (% correct character selections/total selections)	Typing accuracy (% correct character selections/total selections)	Typing accuracy (% correct character selections/total selections)

**Additional dependent variables**	Number of correct letter selections, correct backspace selections, and incorrect selections Characters per minute (total CPM, correct CPM for letters and backspace, correct CPM for letters only) User experience questionnaire responses	Number of correct letter selections, correct backspace selections, and incorrect selections Characters per minute (total CPM, correct CPM for letters and backspace, correct CPM for letters only) User experience questionnaire responses	Number of correct letter selections, correct backspace selections, and incorrect selections Characters per minute (total CPM, correct CPM for letters and backspace, correct CPM for letters only) User experience questionnaire responses	Number of correct letter selections, correct backspace selections, and incorrect selections Characters per minute (total CPM, correct CPM for letters and backspace, correct CPM for letters only) User experience questionnaire responses SSVEP trial length

**Calibration task**	Communicator 5 Gaze Interaction Calibration with 9 target locations	Communicator 5 Gaze Interaction Calibration with 9 target locations	EyeX calibration followed by SSET calibration (20 three-second trials of eye tracking data collected for each of four onscreen targets)	SSBCI calibration (20 six-second trials of SSVEP data collected for each of four LED panel targets positioned at edges of screen)

**Copy-spelling task**	Copy five five-letter English words	Copy five five-letter English words	Copy five five-letter English words	Copy five five-letter English words

**Copy-spelling character selection**	Participant selects a box by gazing steadily at it for 1.2 seconds. Each character selection requires three box selections (large group, small group, and individual character).	Participant selects a box by gazing steadily at it for 2.5 seconds. Each character selection requires three box selections (large group, small group, and individual character).	Participant selects a box by gazing at it for two seconds. Shuffle Speller queries the user as many times as necessary, rearranging the characters among boxes, until one character’s probability exceeds 85%.	Participant selects a box by gazing at the neighboring LED panel for the duration determined by the adaptive trial length feature. Shuffle Speller queries the user as many times as necessary, rearranging the characters among boxes, until one character’s probability exceeds 85%.

### Materials

In all conditions, a 21.5-inch monitor was mounted on a VarioFloat floor stand mount (Rehadapt, Kassell, Germany) and positioned in front of the participant at a distance of approximately 30 inches. The additional hardware and software used in each condition is described below.

**FIGURE 1:**
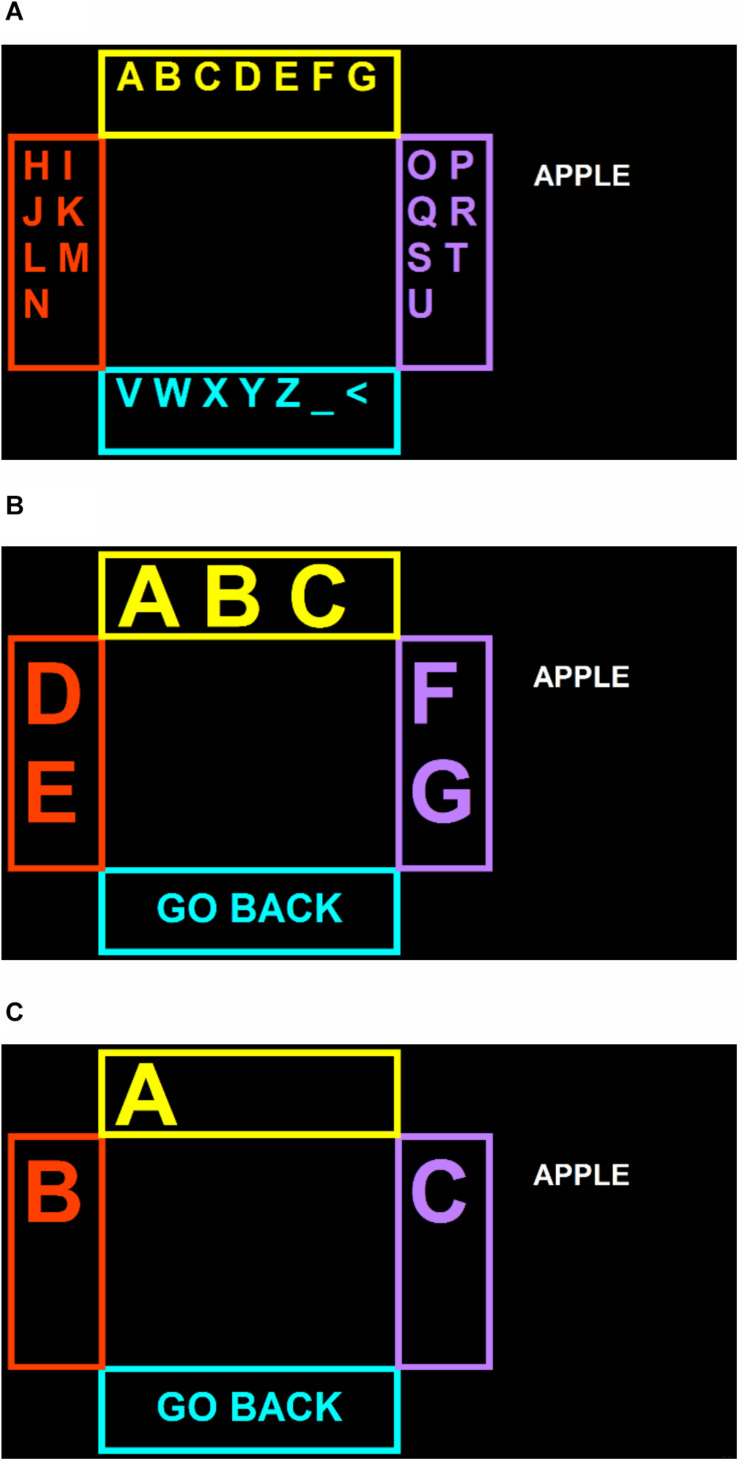
Typing layout and steps **(A-C)** for baseline (C1.2) and C2.5 conditions.

In the baseline condition (C1.2) and in the modified Communicator condition (C2.5) of the comparison phase, participants used a system similar to the eye-tracking SGDs currently available to people with SSPI. Eye tracking data were collected with a PCEye Mini (Tobii Dynavox, Pittsburgh, PA) attached to the monitor, using the Gaze Interaction software packaged with Communicator 5. The Track Status tool in Communicator 5 was used to guide proper monitor positioning for eye tracking. During copy-spelling tasks, the screen displayed a three-step spelling page set created in Communicator 5 and designed to resemble the appearance of Shuffle Speller (Figure 1; page set available at https://www.mytobiidynavox.com/psc/communicator/84098 or by request; instructional video available as Supplementary Material). Four boxes, each a different color and with dimensions of 6° × 18° or 15° × 7° visual angle, were centered at the top, bottom, left, and right edges of the keyboard area, with an area on the right side of the screen displaying a target word and the typed string. Box locations were determined based on participants’ gaze trajectory plots from the pilot study, as well as their successful use in the previously reported study with healthy control participants (Peters et al., 2018a). Box colors were chosen to be maximally dissimilar from one another while maintaining a high contrast ratio against a black background. Letters of the alphabet, plus characters representing space (_) and backspace (<), were arranged in groups within the boxes. Characters were presented in Arial font with visual angles ranging from 1° to 4°. Participants were asked to type by first selecting a large group of letters (Figure 1A), then a smaller group from within that group (Figure 1B), and finally an individual letter (Figure 1C). Letter arrangements were consistent throughout the typing task (i.e., the letters A through G were always displayed in the top box on the first screen, and were always arranged in the same configurations in the subsequent steps, as in Figure 1). A “go back” button allowed participants to return to the previous screen without typing a letter if the wrong group was selected. This typing procedure is similar to that used in the “large key” typing layouts available in many current SGD software packages, with the intention of providing larger targets to users who have difficulty making accurate selections with eye tracking. The custom page set designed for this experiment featured fewer buttons than typical large key page sets, which reduced functionality (e.g., by removing buttons for word prediction, stored phrases, or navigation to other pages), but provided a simplified interface with larger targets.

**FIGURE 2:**
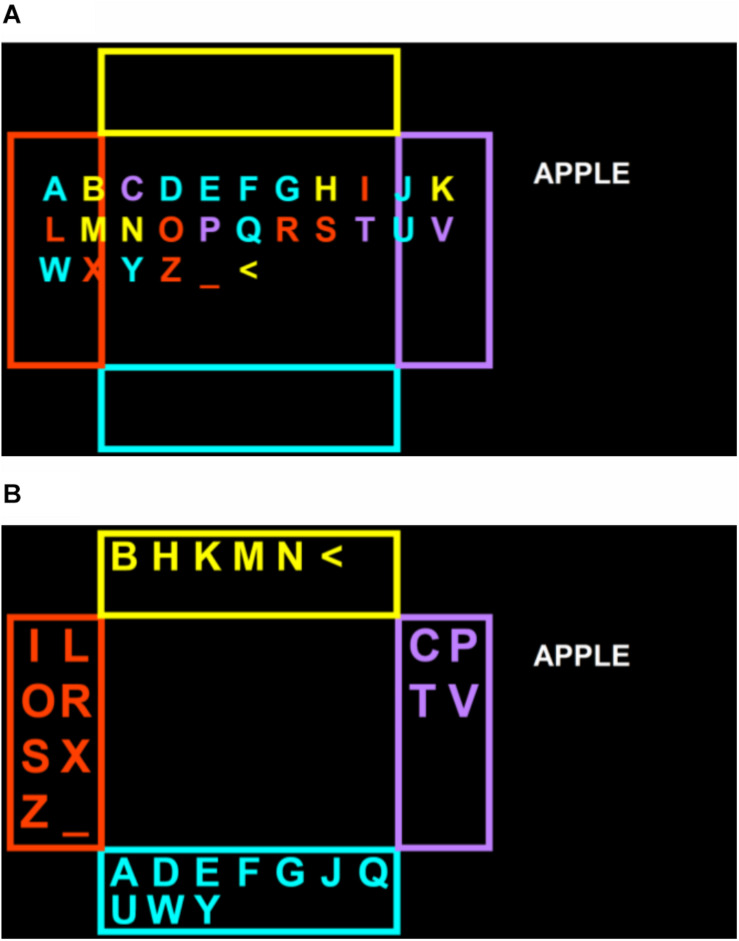
Typing layout and steps for SSET and SSBCI conditions, showing letters before **(A)** and after **(B)** partitioning.

The two remaining comparison phase conditions featured Shuffle Speller, which was presented using MATLAB (MathWorks, Natick, MA), and is described in detail in previous work (Higger et al., 2016; Peters et al., 2018a). In the SSET condition, eye tracking data were collected with an EyeX (Tobii Dynavox, Pittsburgh, PA) at a sampling rate of 60 Hz, using custom MATLAB software and GazeSDK (Tobii Dynavox, Pittsburgh, PA). The “position guide” tool within the EyeX software was used to check for proper monitor positioning, and participants completed a standard EyeX calibration before beginning the Shuffle Speller calibration task. In copy-spelling mode, Shuffle Speller was configured with the same box sizes and general screen layout (Figure 2) as the Communicator 5 page set used in the C1.2 and C2.5 conditions. Prior to the first query for each selection, the characters appeared in alphabetical order (with space and backspace characters at the end) in the center of the screen (Figure 2A) for 5 s before being “shuffled” among the boxes (Figure 2B; instructional video available as Supplementary Material). For each subsequent query until a selection was made, the characters would pause for 2 s before re-shuffling. Character arrangements are determined based on a combination of a mutual information-based alphabet partitioning method, user calibration data (indicating the reliability of user responses to each stimulus), and previous user inputs (Higger et al., 2016; Peters et al., 2018a). Letters changed colors prior to each shuffling to indicate their next box location.

In the SSBCI condition, EEG data were collected using g.BUTTERFLY active electrodes (g.tec, Schiedlberg, Austria) positioned in a non-slip elastic headband (Conair Corp., East Windsor, NJ) at approximate locations over O1, Oz, and O2. Oz was positioned approximately 3cm above the inion, with O1 and O2 approximately 5% of head circumference away on either side. Fpz was used as a ground, and a reference electrode was clipped on the right earlobe. EEG data were sampled at 256 Hz, recorded with a g.USBamp amplifier (g.tec, Schiedlberg, Austria), and were visually inspected for data quality before each BCI task. SSVEP responses were stimulated with LED arrays (each with 1.6° × 2.4° visual angle) secured to the monitor next to each target box in the Shuffle Speller interface. Each array included 25 surface mount LEDs (Kingbright, City of Industry, CA) arranged in a 5 × 5 square, and was attached to the monitor using Dual Lock fasteners (3M, St. Paul, MN) or a custom car-and-rail system (Peters et al., 2018a). Arrays flashed at different frequencies (8.0, 9.7, 11.3, and 13.0 Hz), such that each target elicited a distinct, classifiable SSVEP response (instructional video available as Supplementary Material). The same Shuffle Speller layout (Figure 2) and character distribution strategy were used for both the eye tracking and BCI conditions. Pre-shuffle delay times were the same as in the SSET condition.

### Procedure and Tasks

All data collection visits took place in quiet rooms at participants’ homes, with participants in a reclining position in bed. The study began approximately 6 months after the completion of the pilot study. Data collection visits were conducted weekly, with a consistent day of the week, start time, and visit duration for each participant. Each baseline data-collection visit consisted of a single copy-spelling session, while each visit during the comparison phase included two copy-spelling sessions to reduce the time and resources required to complete the experiment. Each copy-spelling session consisted of a calibration task and a copy-spelling task, as well as a user experience (UX) questionnaire. All calibration and copy-spelling tasks were preceded by a short instructional video to remind the participant of the task procedures. The videos (available online as Supplementary Materials) ensured consistent instructions across sessions and easy discrimination among conditions.

#### Calibration Tasks

The baseline (C1.2) and C2.5 conditions used the Calibrate feature within the Gaze Interaction settings in Communicator 5. Participants were asked to track a target as it moved around the screen, fixating on it as it paused in each of nine locations.

For the SSET condition, participants first attempted to calibrate the EyeX using the calibration task in the EyeX software, fixating on a series of seven small dots appearing at different locations on the screen. If they were unable to do so (typically due to inconsistent eye tracking or difficulty fixating on targets at the edges of the screen), a researcher or caregiver completed that initial calibration under the same lighting conditions and at the same distance from the screen as the participant. Next, the participant completed the SSET calibration. Four square targets with dimensions of 0.8° × 0.8° visual angle were displayed in the center of the top, bottom, right, and left edges of the screen. In each trial, one of the squares would turn red to mark it as the next target, then green as the participant’s eye position was recorded while gazing at the designated target. A short beep alerted participants to the appearance of the next target. Participants were instructed to look at the green target square in each trial. Eye tracking data were recorded for 3 s in each trial. Calibration included 20 trials for each of the four targets, and lasted approximately 8 min.

In the SSBCI condition, calibration targets consisted of the four LED arrays positioned at the edges of the screen. The target for each trial was indicated by the appearance of one of the four colored boxes used in the Shuffle Speller interface. Participants were instructed to look at the flashing light next to the target box during each trial. SSVEP data were recorded for 6 s in each trial. Calibration included 20 trials for each of the four targets, and lasted approximately 12 min. EEG features were constructed as canonical correlation analysis (CCA) scores from a segment of time between all EEG channels and the first and second harmonics of the stimulation frequency. A kernel density estimate (KDE) was constructed, per user and stimulation frequency, which allowed our model to encapsulate variability in SSVEP response strength. This model furnished a likelihood score per stimulation, allowing for a principled Bayesian update of our confidence in each target letter. Additionally, it provided a confusion matrix which quantified how often stimulation conditions were misclassified in a particular user, which was then used to optimize the alphabet partitioning to gain the most information from each user query (Higger et al., 2014). Similarly, to choose the trial length for SSVEP stimulation (i.e., the duration of LED flickering for each trial), trials were truncated to various lengths and expected information transfer rate was computed. Note that we use Nykopp’s information transfer rate, which does not impose strong symmetry assumptions on the confusion matrix as in the historically prevalent BCI definition (Nykopp, 2001; Kronegg et al., 2005; Peters et al., 2018a).

#### Copy-Spelling Tasks

During each copy-spelling session, participants had the opportunity to copy-spell a list of five words. A total of 65 common, five-letter English words with similar frequency of use were drawn from the SUBTLEXus dataset (Brysbaert and New, 2009). A set of 26 comparable word lists, each consisting of five words, was generated using a web-based list randomizer. A word list was randomly selected (without replacement within participants) for each session using a web-based random number generator (both randomization tools available at www.random.org). Target words were displayed one at a time on the right side of the screen, as shown in Figures 1, 2. Copy-spelling sessions were limited to 20 min to avoid participant fatigue and frustration, and were terminated if no correct selections were made in the first 5 min of typing. If four incorrect selections were typed consecutively, or if Shuffle Speller was unable to recognize user input in too many consecutive trials, the task advanced to the next word on the list.

In the baseline (C1.2) condition, participants made box selections using a 1.2-s dwell click, with visual feedback to indicate progress toward a click. In the C2.5 condition, the dwell click duration was extended to 2.5 s. In both the C1.2 and C2.5 conditions, three box selections were required to select each character, as described in the “Materials” section and illustrated in Figure 1. In the SSET condition, participants were instructed to look at the box containing their letter in each query. After each box selection, the characters were rearranged as described in section “Materials,” and character probabilities were updated using a generative model based on recursive Bayesian inference (Higger et al., 2014, 2016). They pursued their target and attempted to select the appropriate box through as many queries as necessary for one character’s probability to exceed a confidence threshold of 85%, at which point the character was typed. The typing method was similar for the SSBCI condition, except that participants looked at the LED array next to the box containing their target letter instead of at the box itself. In both Shuffle Speller conditions, character selection was based on recursive Bayesian updates rather than input detection alone.

In all conditions, each selected letter was spoken aloud in synthesized speech presented through the laptop speakers. Selected letters also appeared in the dashboard area on the right side of the screen, beneath the target word. Correctly typed letters were displayed in green, while incorrect letters were displayed in red. Participants were instructed to correct any typing errors by selecting the backspace character.

#### User Experience Questionnaire

An adapted UX questionnaire was used to solicit participant ratings of workload, comfort, and satisfaction with system use. Three satisfaction questions focused on typing accuracy, typing speed, and overall satisfaction. The questionnaire was designed specifically for use with individuals with SSPI, with response options presented using partner-assisted scanning with both speech and visual analogs (Peters et al., 2016). Response options for all questions were in the format of a labeled, 7-point Likert scale.

### Dependent Variables, Data Collection, and Analysis

Four dependent variables were measured: typing accuracy, typing speed, SSBCI trial length and UX responses. The primary dependent variable was typing accuracy during copy-spelling tasks, calculated as the percentage of correct character selections out of total selections in each session. The selection of backspace to correct an error was considered a correct response for purposes of calculating accuracy. Because both participants demonstrated frequent errors and error corrections, an additional dependent variable included the number of selections made within three categories: correct letter selections, correct backspace selections (to delete a letter selected in error), and incorrect selections. Although the appropriate selection of backspace was considered correct for calculating accuracy, it did not advance the participant closer to completing the typing task and was thus considered separately. For this reason, an additional accuracy metric was calculated, using the percentage of correctly typed letters out of total selections (i.e., backspace selections were not included as correct selections). Characters per minute (CPM) was chosen as a dependent variable to measure typing speed. Due to the high number of errors and error corrections, CPM was calculated in three different ways to more clearly illuminate differences among the conditions: (1) total CPM (total number of selections, including all correct and incorrect selections, divided by total typing time), (2) correct CPM including correct letter and backspace selections (total number of correct selections divided by total typing time), and (3) correct CPM including only correct letters (total number of correct letter selections divided by total typing time). Other dependent variables included SSBCI trial length (chosen automatically by Shuffle Speller based on SSVEP calibration data) and UX questionnaire responses.

Each character selected during copy-spelling tasks was recorded and classified as a correct letter selection, a correct backspace selection, or an incorrect selection. Total typing time, excluding breaks between words, was recorded for each copy-spelling session. Typing accuracy and the three measures of typing speed were calculated using Excel (Microsoft, Redmond, WA). For typing accuracy, the primary dependent variable, data were plotted in Excel and visual analysis was used to assess behavior within and across conditions, including level, trend, and variability (Ledford et al., 2018). Measures of central tendency and variability were calculated using Excel for accuracy, SSBCI trial length, and UX questionnaire responses. Data were stored and managed using REDCap electronic data capture tools (Harris et al., 2009, 2019) hosted at OHSU. All copy-spelling sessions were recorded with a digital video camera.

### Procedural Fidelity and Data Collection Reliability

A procedural checklist, including the essential steps for each condition, was used to ensure that the independent variable (copy-spelling spelling condition) was consistently controlled throughout the experiment (Ledford and Gast, 2014). Procedural fidelity was measured for 25–33% of data collection visits in each phase (including the baseline phase), selected using a random number generator. As the primary researcher set up the typing system, interacted with the participant, and implemented the tasks described above, an observer familiar with the experiment monitored and recorded completion of the essential steps on the checklist.

Data collection reliability was assessed for the same set of randomly selected visits. A researcher who was not present for data collection reviewed the video recording of the copy-spelling session, recorded data and calculated the dependent variables, and compared results with those recorded during the session.

## Results

With participants experiencing SSPI secondary to late-stage ALS, it is not uncommon for challenges to arise during an experimental paradigm. As such, several adjustments were made during the course of the study. For Participant 1, the C2.5 condition was discontinued after week 8, after four consecutive sessions with 0% accuracy. Participant 1 had a substitute paid caregiver on the day of the data collection visit in week 12, and reported being unusually fatigued due to the change in his daily routine. Two initial baseline phase sessions for Participant 2 were discarded prior to analysis due to a change in monitor positioning. At the end of the second session, Participant 2 suggested the monitor be positioned such that his gaze was centered among the four boxes in the keyboard area, instead of in the center of the screen. This position appeared to improve his performance, and was maintained for all conditions throughout the rest of the experiment. Only the three baseline data points collected after this change were included in data visualization and analysis for Participant 2. Data collection for Participant 2 was canceled in week 3 due to researcher illness. Participant 2 reported high levels of fatigue during data collection in weeks 12 and 13 and his paid caregiving staff reported that he was ill, but he said he felt well enough to participate in the experiment and did not wish to cancel.

**FIGURE 3:**
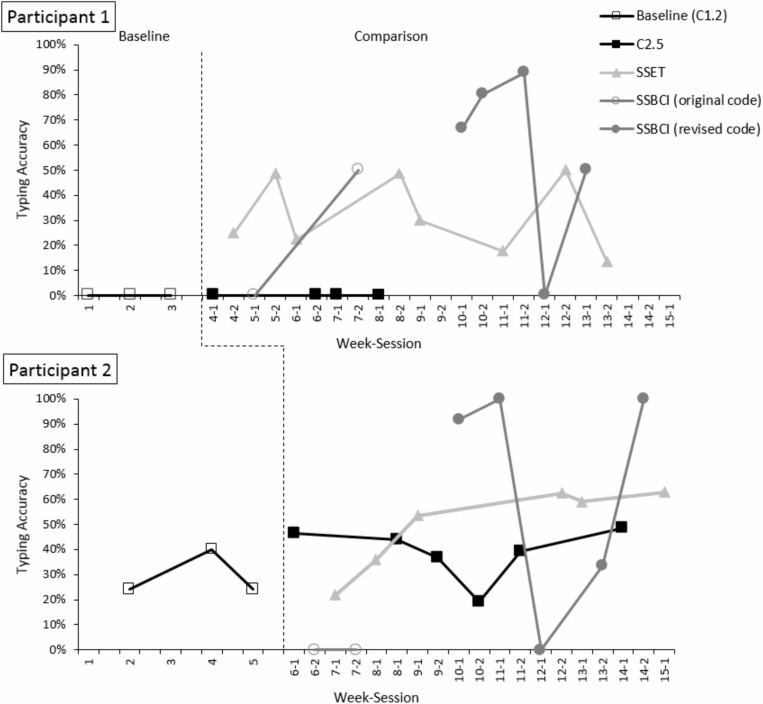
Typing accuracy (with backspaces included) in baseline and comparison phases. The dashed line indicates the transition between the baseline and comparison phases.

Following week 7 of the study, an error was discovered in the SSBCI code that generated classifiers based on calibration data. SSBCI condition sessions were suspended until the code was revised, resuming in week 10. Although all SSBCI sessions are represented in the plot of typing accuracy data (Figure 3), those conducted prior to the code revision were excluded from visual analysis and calculations of central tendency, and are represented separately in the figure. The comparison phase was re-started in week 8 and continued until data had been collected in five copy-spelling sessions for each condition within the same phase.

Participant performance in the SSBCI condition was highly variable (see Figure 3). Although EEG signals were visually inspected for quality before each calibration and copy-spelling session, the software did not provide real-time display of the data during the tasks. After data collection, EEG data were visually inspected for overall channel quality using EEGlab version 14.1.2 (Delorme and Makeig, 2004). After applying a bandpass filter of 1–70 Hz, each dataset was broken into 10 s intervals and reviewed for the presence of non-neural activity. In SSBCI copy-spelling sessions with low accuracy (session 12-1 for P1 and sessions 12-1 and 13-2 for P2), one or more channels was found to be of poor quality, with intermittent, high-amplitude non-EEG signals. It is likely that an equipment problem or other data-collection issue, participant-related factors (illness or fatigue, as described above), or some combination of both, contributed to participants’ poor performance in these sessions.

### Accuracy

Typing accuracy (including correct backspace selections) for both participants is displayed in Figure 3. Participant 1 was unable to make any correct selections in either the baseline (C1.2) or C2.5 condition. He completed eight sessions with SSET and five sessions with SSBCI (after the code revision). There were immediate increases in accuracy with the introduction of the SSET condition in week 4 (to 25.0%), as well as the SSBCI condition in week 10 (to 66.7%). His performance improved over the first three SSBCI sessions (to a maximum of 88.9%), followed by a sharp drop-off (to 0%) in week 12. Poor signal quality, as well as the change in Participant 1’s caregiving routine (causing increased fatigue) on the day of this visit, may have affected his performance. Participant 1’s mean accuracy for SSET was 31.9%, with a range 13.3–50.0%. His mean SSBCI accuracy was 57.0%, with a range of 0.0–88.9%. Variability was high for both Shuffle Speller conditions, but particularly for SSBCI. Overall, the data suggest that both SSET and SSBCI allowed higher typing accuracy compared to C2.5 for Participant 1, with eight demonstrations of an effect for SSET and four for SSBCI. The difference between SSET and SSBCI is less clear, as only three of five SSBCI sessions were superior to SSET. The decline to 0% accuracy with SSBCI in session 12-1 was an unexpected change, and although accuracy increased to 50.0% the following week, it is possible that the data show a decelerating trend. Of note, Participant 1 achieved his highest accuracy with SSBCI (88.9%; session 11-2) during the same visit in which he demonstrated very low accuracy with SSET (17.6%; session 11-1). His best performance with SSET (50.0% in session 12-2) occurred on the same day as his worst performance with SSBCI (0% in session 12-1).

Average baseline accuracy for Participant 2 was 29.0%, with a range of 24.1–40.0%. Accuracy was similar with an increased dwell time in the C2.5 condition, with mean 39.0% and range 19.2–48.5%. In the SSET condition, Participant 2’s mean accuracy was 49.3%, with range 21.9–62.9% and an increasing trend. Mean SSBCI accuracy was 65.0%, with a range of 0–100%. Like Participant 1, Participant 2 experienced an abrupt drop-off in SSBCI performance, to 0%, in week 12. Again, there were both participant-related factors (fatigue and illness) and signal quality problems that may have affected performance for this session. His SSBCI accuracy improved to 33.3% in week 13 and then to 100% in week 14 as he returned to health. Variability was relatively low for the last four SSET sessions, and high for the SSBCI condition. Participant 2 demonstrated higher typing accuracy with SSET than C2.5, with four demonstrations of an effect. Differences between SSBCI and the other conditions are less clear. Participant 2’s performance in three of five SSBCI typing sessions was superior to his best performance for either the C2.5 or SSET condition. On days when Participant 2 performed poorly with SSBCI (in weeks 12 and 13), his accuracy with SSET remained relatively stable.

**FIGURE 4:**
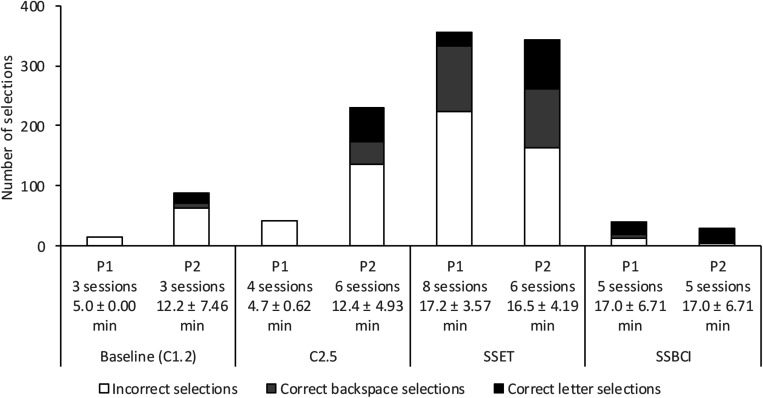
Total number of incorrect selections, correct backspace selections, and correct letter selections for each condition and participant. Labels indicate the number of sessions completed by each participant in each condition, and the mean ± standard deviation of session length. P1, Participant 1; P2, Participant 2.

It should be noted that, for both participants, the correct selection of the backspace character accounted for a large number of the correct selections used to calculate accuracy. Figure 4 displays the total number of correct letter selections, correct backspace selections, and incorrect selections, aggregated across all copy-spelling sessions for each condition. The number of sessions completed in each condition varied, as did the mean session length (as a result of stoppage criteria); number of sessions and mean ± standard deviation for session length are indicated in the bar labels in Figure 4. Although a total of 36.9% of Participant 1’s selections in the SSET condition were correct, the majority (82.4%) of those were backspace selections rather than correct letter selections. By contrast, 70.0% of his SSBCI selections were correct, with the majority (75.0%) being correct letter selections. He also had seven correct backspace selections in the SSBCI condition, allowing him to fix seven of 12 incorrect selections. Participant 1 typed far fewer characters overall using SSBCI than SSET, but the number of correct letter selections was very similar. Participant 2 had a higher percentage of total correct selections for both C2.5 and SSET compared to baseline, but backspace selections made up 39.4% of C2.5 correct selections and 54.1% of SSET correct selections. Participant 2’s total number of selections was also much lower for SSBCI than the other conditions, but with a higher total percentage of correct selections (89.3%) and a higher proportion of those being correct letter selections (96.0%).

**FIGURE 5:**
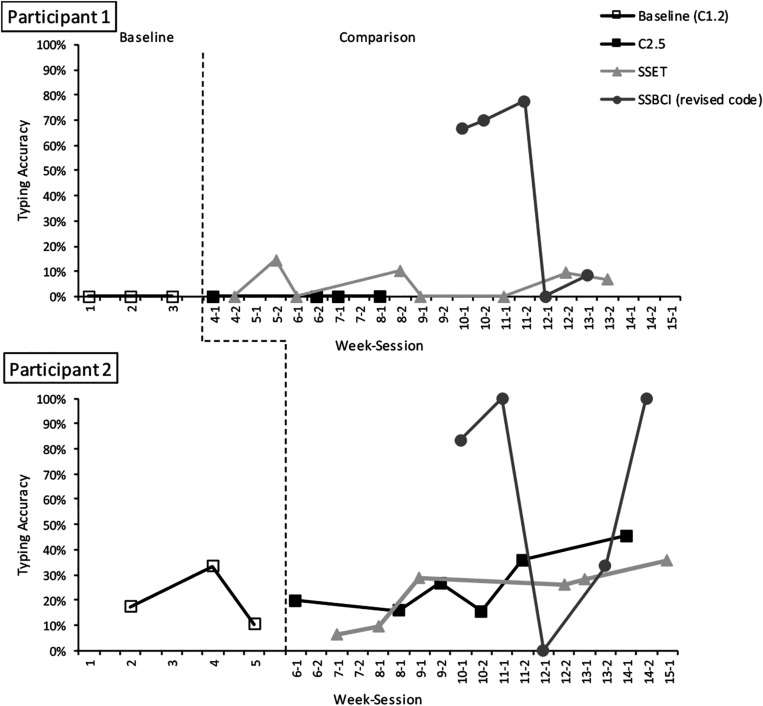
Typing accuracy (for correct letter selections only, excluding backspaces) in baseline and comparison phases. The dashed line indicates the transition between the baseline and comparison phases.

Because of the large number of errors and backspace selections, accuracy was recalculated to reflect the percentage of correct letter selections (excluding backspace selections) out of total selections. Results of this alternate accuracy metric are displayed in Figure 5. SSBCI selection remains low on days affected by poor signal quality and participant fatigue or illness. With the exclusion of backspace selections, there were considerable reductions in accuracy for SSET for Participant 1 and for C2.5 and SSET for Participant 2, reflecting the high error rate in these conditions. With accuracy calculated using this method, Participant 1 had a mean of 5.1% (range 0.0–14.5%) for SSET and 45.0% (range 0.0–77.8%) for SSBCI. Both Shuffle Speller conditions still show improvement compared to C2.5, with four demonstrations of effect for both SSET and SSBCI. Three of five SSBCI sessions showed superior performance compared to SSET. With correct backspace selections excluded, Participant 2 has a slightly lower baseline accuracy of 20.0% (range 10.3–33.3%). In the comparison phase, he demonstrated mean accuracies of 27.8% (range 15.4–45.5%) for C2.5, 22.3% (range 6.3–35.5%) for SSET, and 63.3% (range 0.0–100.0%) for SSBCI. SSBCI is superior to both C2.5 and SSET in three of five sessions, but there is no longer a demonstration of effect for SSET compared to C2.5.

### Characters Per Minute

**FIGURE 6:**
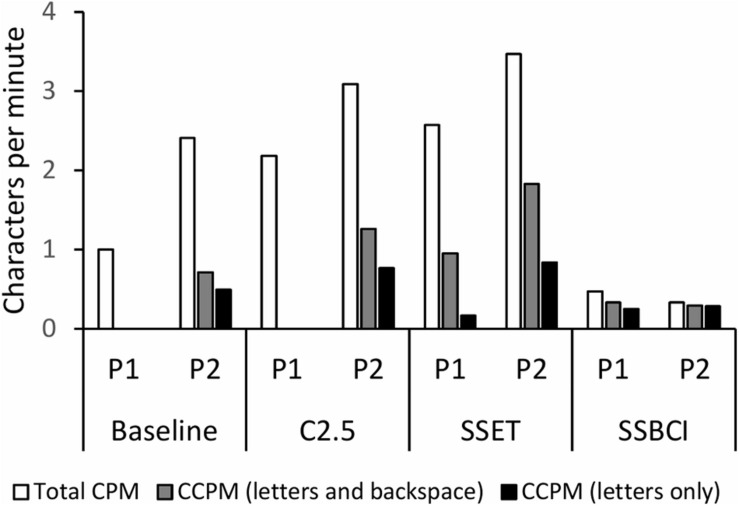
Total characters per minute (CPM), correct characters per minute (CCPM) with correct letter and backspace selections, and CCPM with correct letter selections only for each condition and participant. P1, Participant 1; P2, Participant 2.

The three measures of typing speed for each condition and participant are displayed in Figure 6. Differences among values for total CPM, correct CPM including letters and backspace, and correct CPM including only letters reflect the varying proportions of correct selections represented in Figure 4. Although total CPM for SSBCI was much lower than for the other conditions, it was very similar to the correct CPM values for SSBCI, as we might expect given the relatively high proportion of correct selections achieved by both participants within that condition. Of the three typing speed measures, correct CPM including only correct letter selections is the best indicator of the actual communicative effectiveness for each condition. Participant 1 demonstrated his highest correct CPM for letters with SSBCI. Participant 2 selected more correct letters per minute with both C2.5 and SSET compared to SSBCI, but at a cost of far more frequent errors and required error corrections, as shown in Figure 4.

### SSBCI Trial Length

Trial lengths across five SSBCI copy-spelling sessions averaged 5.30 s (SD 0.425) for Participant 1 and 4.81 s (SD 1.723) for Participant 2.

### User Experience

Participant 1 chose not to complete the UX questionnaire, as his yes/no responses required substantial time and effort due to his slow and inconsistent movements, and answering the questions would have added considerably to the length of his data collection sessions and his resulting fatigue. Participant 2’s questionnaire responses are displayed in Table 3. Lower scores are more favorable, with 1 representing no workload, no discomfort, and highest satisfaction, and 7 representing extreme workload, extreme discomfort, and lowest satisfaction. Across all categories, Participant 2 rated all three conditions from the comparison phase more favorably than the baseline condition. UX ratings for C2.5 and SSET were similar for all questions. Participant 2 rated the SSBCI condition best for both workload and comfort, with mean ratings of 2.4 and 2.0, respectively. His mean ratings for SSBCI accuracy, speed, and overall satisfaction were slightly better those for C2.5 and SSET, but with greater variability. For the three sessions in which his SSBCI typing accuracy was 92–100%, he gave good ratings (1, 2, or 3) for all satisfaction questions. For the two sessions in which his accuracy was 0–33%, he gave poor ratings (5, 6, or 7) on the same questions.

**TABLE 3:** Mean (SD) user experience questionnaire responses for Participant 2.

	**Baseline (C1.2)**	**C2.5**	**SSET**	**SSBCI**
**Workload**	6.0 (0.00)	3.5 (1.05)	3.2 (1.33)	2.4 (0.55)
**Comfort**	3.7 (0.58)	2.5 (1.05)	2.8 (2.14)	2.0 (0.00)
**Accuracy satisfaction**	5.0 (1.00)	4.5 (1.64)	4.8 (1.17)	4.0 (2.83)
**Speed satisfaction**	5.7 (0.58)	3.8 (0.98)	4.0 (1.26)	3.8 (2.17)
**Overall satisfaction**	5.7 (0.58)	4.8 (1.33)	4.5 (1.64)	3.8 (2.59)

At the end of the study, each participant was asked which system he preferred, and what he liked or disliked about each system. They could select from a list of suggested answers using yes/no responses, or provide their own answers using partner-assisted scanning (Participant 1) or an SGD (Participant 2). Participant 1 preferred the SSBCI system because typing was more accurate, but reported that the flashing LEDs caused some eye discomfort. Participant 2 also preferred SSBCI, reporting that it was more comfortable to use than eye tracking and allowed him to “feel in control,” though he disliked the flashing LEDs. He was frustrated with the eye tracking condition because his “glasses interfere[d] with the system,” and reported that SSET selections often felt “random.”

### Procedural Fidelity and Data Collection Reliability

Procedural fidelity was assessed for one visit in the baseline phase and two visits in the comparison phase for each participant, for a total of 33% of baseline sessions and 20% of alternating-treatments sessions. Both treatment fidelity and data collection reliability were 100% in both phases.

## Discussion

There are few published reports of experimental studies on non-invasive BCI and AAC performance of individuals with late-stage ALS and minimal volitional movement. It can be challenging to include participants with this level of disability in research. BCI research, in particular, often involves those with less severe impairments, or healthy participants. Despite the difficulties with data collection described above, there is significant value in reporting results from PALS with this level of impairment. The information gained from empirical studies such as this one will support crucial clinical decision making for individuals with late-stage ALS who are not well served by existing assistive technology options. Research exploring BCI as a potential access method for people with SSPI has often failed to address the visual demands of the system or the visual skills of users, so this study offers a unique contribution.

The two PALS who participated in this study presented with visual impairments including reduced ocular motility, reduced visual acuity, ptosis, double vision, and light sensitivity, and demonstrated poor performance using commercially available eye tracking software. Shuffle Speller appears to hold some promise as a typing interface for this population, whose input signals for computer control may be inconsistent or difficult to detect. Participant 1 was unable to type any correct characters in either of the standard eye tracking conditions (baseline or C2.5), but achieved accuracies of up to 50% with SSET and 89% with SSBCI. Participant 2 also had higher maximum accuracies for the Shuffle Speller conditions than the standard eye tracking conditions, typing with up to 63% accuracy for SSET and 100% accuracy for SSBCI. However, participants’ typing accuracy for both Shuffle Speller conditions was variable, particularly for SSBCI, indicating that additional improvements may be needed to optimize performance for this user population.

These results also show the potential of SSVEP BCI as an assistive technology access method for individuals with late-stage ALS and visual impairments. Each participant demonstrated high typing accuracy in three of five typing sessions in the SSBCI condition, and a single session for each served as an extreme outlier preventing a cleaner comparison. Although typing speed was much slower with SSBCI than in the other conditions, both participants made a higher proportion of correct selections with SSBCI, and preferred it over the eye tracking conditions due to the higher accuracy and feeling of control. Participant 2 rated the SSBCI condition highest for both workload and comfort, though his satisfaction ratings varied considerably along with his performance. Both participants reported that the flashing LED panels in the SSBCI condition caused mild discomfort. Although they still preferred SSBCI over the other conditions which did not involve flashing LEDs, these comments highlight the importance of exploring alternative stimulation methods to increase comfort during SSVEP BCI use for individuals with disabilities.

The two participants in this study achieved maximum SSBCI accuracies of 89 and 100%, with mean accuracies across sessions of 57.0 and 65.0% and substantial performance variability. It is difficult to make comparisons between this and other studies, as BCI studies involving PALS with both extremely limited volitional movement and visual or oculomotor impairments are rare, and investigations of SSVEP BCI in this population have typically not involved typing tasks. Hwang et al. (2017) tested a four-class SSVEP BCI with five PALS meeting this description, with mean within-subject classification accuracies on a simple multiple-choice task ranging from 53.9 to 87.5%, and varying levels of performance for the same participants on different days. Another SSVEP BCI study included three individuals with late-stage ALS and minimal movement, two of whom presented with either inconsistent ocular motility or a complete inability to move the eyes. Each participant was asked to attend to targets presented using one, two, or three flashing LEDs. Mean accuracy on an attend/ignore task was 84.3%, but no communication task was attempted as part of the experiment. The PALS with the most restricted eye movement demonstrated some potential for answering yes/no questions by attending to or ignoring a single LED stimulus over repeated trials, but this was not systematically explored (Okahara et al., 2018). In another study, three PALS with late-stage ALS and “slow eye movements” attended to a flashing stimulus to activate an emergency call system. During a series of 4 weekly experimental sessions, one participant experienced a “sudden drop” in system performance, similar to the sudden drop described in the current study (Lim et al., 2017). The results presented for our SSBCI condition, involving more LED stimuli and full typing capability than these earlier studies, show additional promise for the use of SSVEP BCI with this population with significant, and variable, disabilities.

Some non-invasive BCI studies (typically involving P300 spellers) have reported higher mean accuracies among participants with ALS, such as (Speier et al., 2017; Guy et al., 2018; Ryan et al., 2018). Overall, studies involving non-invasive BCI for PALS tend to average around 70% accuracy, with significant heterogeneity (Marchetti and Priftis, 2015). Most studies involve PALS with less severe disability (typically represented by higher ALSFRS-R scores), or a group of PALS with a wide range of disability levels, and very few have specifically included those with concomitant visual impairments. In fact, participants’ visual skills are rarely described in reports of visual BCI studies, though they do appear to affect performance. For example, McCane et al. (2014) noted that, of 25 participants with ALS, the eight who performed with <70% accuracy on a P300 speller typing task all presented with visual impairments. BCI studies frequently report ALSFRS-R scores as measures of physical impairment, but even among PALS with ALSFRS-R scores of 0 there is a wide range of ability when it comes to AAC access. The ALSFRS-R does not assess eye movement or other small movements that could be used to activate a switch, both of which can be crucial for communication in late-stage ALS, nor does it assess visual skills for interacting with a visual interface. In short, more evidence is needed on the effectiveness of non-invasive BCI systems for PALS with the most severe physical, oculomotor, and visual impairments, and more detailed participant description is vital for allowing better comparison among studies and facilitating eventual clinical implementation.

Typing performance for this population might be improved with the implementation of a hybrid system incorporating both eye tracking and SSVEP as control signals. Poor SSBCI performance in weeks 12 and 13 for both participants suggests that fluctuations in user state (e.g., fatigue or illness) and in the quality of data collection in the home environment may present challenges to the use of SSVEP for typing in this population. A hybrid system could be designed to take advantage of the most reliable control signal, or combination of signals, based on calibration data, making performance more resilient to changes in user state (Müller-Putz et al., 2015). In future iterations of the system, it will be important to include real-time display of signals so the equipment operator can more quickly recognize and correct poor signal quality that may otherwise result in poor performance. Customized user interface design may be another avenue for improving performance. Modified Shuffle Speller layouts (with a different number or configuration of boxes), or changes to animation speeds, font sizes, or colors, adapted to an individual user’s visual skills and deficits, may increase comfort and ease of use as well as typing accuracy and efficiency. Future developments will also include integrating flickering stimuli into the typing interface for the SSVEP Shuffle Speller, instead of using external LED stimuli.

The single-case research design employed in this experiment proved useful for exploring performance over time in a small sample of adults with SSPI. These research designs can demonstrate intervention effects or compare effects between treatments with a small number of participants. This may be valuable for investigating BCI and other assistive technologies, given that target users for these systems are often individuals with rare conditions who may be difficult to recruit for study participation. The repeated measurements integral to single-case research design also provide an opportunity to identify variability across participant responses, and to understand how and why these variations occur. As reported in the current results, the performance of study participants in this population may be highly variable, likely related to changes in alertness or attention due to factors such as fatigue, illness, or medications. Many BCI studies involving participants with disabilities report performance data from only a single day, which would not capture the full range of performance for participants with this level of variability. Single-case research designs allow for the identification of trends (such as a learning effect) and outliers within individual participants as well as intervention effects across participants. This methodology may serve as a useful tool as BCI research involving participants with disabilities moves from case studies to more formal hypothesis testing to support evidence-based clinical practice.

Although these results show promise for Shuffle Speller and SSVEP BCI for this population, the number of participants was limited and the variability in the outcome data makes it difficult to draw strong conclusions. The selection of typing accuracy as the primary dependent variable was problematic, as the stoppage criteria (which affected session length and thus number of characters typed) and the variable typing rate allowed by Shuffle Speller resulted in accuracies calculated with a wide range of denominators, from 2 to 80 typed characters. Number of target characters copied may be a more appropriate primary dependent variable for future copy-spelling experiments with individuals with late-stage ALS, as it may be more representative of the potential effectiveness of a system in realistic communication situations.

Future experiments with Shuffle Speller will involve more individuals with late-stage ALS or with other conditions causing SSPI, and may explore hybrid BCI-ET input signals or custom interface designs to improve performance based on each user’s unique visual abilities and limitations. Based on these promising early results, Shuffle Speller as an interface and SSVEP BCI (and hybrid systems incorporating it) appear to have potential to offer improved computer and communication access to a population with few existing assistive technology options.

## Data Availability Statement

The raw data supporting the conclusions of this article will be made available by the authors, without undue reservation.

## Ethics Statement

The studies involving human participants were reviewed and approved by the Oregon Health & Science University (OHSU) Institutional Review Board. The patients/participants provided their written informed consent to participate in this study.

## Author Contributions

BP, SB, BE, MK, DM, BO, SS, DE, and MF-O contributed to the conception and design of the study. MH and FQ created Shuffle Speller. MH, FQ, and TM provided hardware and software support for this experiment. BP created the Communicator 5 page set and wrote the first draft of the manuscript. SD identified target words for copy-spelling. BP, BE, MK, and DM collected the data. BP and SS analyzed the data. MF-O and SS wrote sections of the manuscript. All authors contributed to manuscript revision, and read and approved the submitted version.

## Conflict of Interest

The authors declare that the research was conducted in the absence of any commercial or financial relationships that could be construed as a potential conflict of interest.

## Supplementary Material

Supplementary Video 1 C1.2 and C2.5 Calibration instructions.

Supplementary Video 2 C1.2 Copy-spelling instructions.

Supplementary Video 3 C2.5 Copy-spelling instructions.

Supplementary Video 4 SSBCI Calibration Instructions.

Supplementary Video 5 SSBCI Copy-Spelling Instructions.

Supplementary Video 6 SSET Calibration Instructions.

Supplementary Video 7 SSET Copy-Spelling Instructions.
